# Two co‐inherited novel SNPs in the *MC4R* gene related to live body weight and hormonal assays in Awassi and Arabi sheep breeds of Iraq

**DOI:** 10.1002/vms3.421

**Published:** 2020-12-27

**Authors:** Tahreer M. Al‐Thuwaini, Mohammed Baqur S. Al‐Shuhaib, Frederic Lepretre, Halla Hassan Dawud

**Affiliations:** ^1^ Department of Animal Production College of Agriculture Al‐Qasim Green University Babil Iraq; ^2^ University of Lille Plateau de Genomique Fonctionnelle et Structurale Lille France

**Keywords:** genetic polymorphism, haplotype, *in silico*, production trait

## Abstract

Melanocortin‐4 receptor (*MC4R*) gene plays a key role in the regulation of body weight and energy homeostasis. This study aims to evaluate the association of single nucleotide polymorphisms (SNPs) of the *MC4R* gene with live body weight and hormonal assays in two breeds of sheep that differ in productive performance, Awassi and Arabi. All known coding sequences of the *MC4R* gene were covered in this study. DNA samples from 150 animals (Awassi and Arabi breed) were genotyped by PCR‐single‐strand conformation polymorphism (PCR‐SSCP) to assess their pattern of genetic variation. Concerning exon 1, clear heterogeneity was detected with three different SSCP‐banding patterns. The sequencing reactions confirmed these variations by detecting the presence of the two novel SNPs, 107G/C and 138A/C, and three genotypes, GC, AC and AA. The 107G/C SNP was detected in GC genotype, while the 138A/C was detected on both GC and AC genotypes. The other SSCP‐banding pattern (AA genotype) did not show any detectable unique variation. Both SNPs were closely and strongly linked in both breeds (D' and *r* ^2^ values were 1.00), which signifies that both loci were co‐inherited as one unit. Association analysis indicated that both breeds with GC/AC haplotype showed higher live body weight (37.250 ± 0.790) relative to the GG/AA (30.244 ± 0.968) and CC/CC (47.231 ± 1.230) haplotypes (*p* < .05). Concerning the genotyping of exon 2, only 362 bp showed heterogeneity with a missense mutation, with no significant association (*p* > .05) with the measured traits. In conclusion, the two novel SNPs (107G/C and 138 A/C) were highly associated with live body weight in both breeds. Haplotype analysis confirmed that these two novel SNPs were in strong linkage disequilibrium (LD) and could be used as genetic markers for sheep phenotypic trait improvement.

## Graphical abstract

MC4R gene effect on the regulation of live body weight and hormonal assays in Awassi and Arabi sheep. The sequencing reactions confirmed the presence of the two novels SNP 107G/C and 138A/C in exon 1 of MC4R gene. The two novel SNP (107G/C and 138 A/C) were highly associated with live body weight. GC/AC haplotype showed higher live body weight relative to the GG/AA and CC/CC haplotypes. Haplotype analysis confirmed that these two novel SNP were in strong linkage disequilibrium and could be used as genetic markers for sheep phenotypic traits improvement.

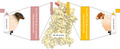

## 1 INTRODUCTION

Body weight in sheep is a pivotal trait for economic breeding and is controlled by multiple genetic loci (Esmailizadeh, 2010). Among these loci, the melanocortin‐4 receptor (*MC4R*) gene plays an important role in the regulation of body weight, energy balance and reproduction in humans and animals (Bakos et al., 2016; Siljee et al., 2013; Zeng et al., 2014). The ovine *MC4R* gene is mapped on chromosome 23 and consists of two exons (Shishay et al., 2019). The *MC4R* gene is involved in the regulation of phenotype expressions of several economic traits in ruminant and non‐ruminant animals. These traits are largely regulated by the binding of the *MC4R* gene‐encoded receptors to four ligands of melanocyte‐stimulating hormone (α‐, β‐ and γ‐MSH) and the adrenocorticotropic hormone (ACTH; Switonski et al., 2013).

The *MCR* family consists of five members (*MC1R* to *MC5R*); each of which has seven transmembrane domains belonging to the G protein‐coupled receptor superfamily (Li & Li, 2006). Out of these five melanocortin receptors, *MC4R* has received more attention. It is expressed in the appetite‐regulating areas of the brain that involve food intake, which can act as a critical mediator between appetite and reproduction in animals (Zandi et al., 2014). Thus, the *MC4R* gene can be a major mediator of leptin effects on food intake and body weight (El‐Sabrout & Soliman, 2018; Hwa et al., 2001; Li & Li, 2006). The effects of the *MC4R* gene variation have been recently implicated in the rapid growth selection program of beef cattle (Prihandini & Maharani, 2019). On the other hand, it has been shown that some of the variations detected in the *MC4R* gene have been associated with economic traits in livestock (Shishay et al., 2019). It has been recently reported that the *MC4R* gene polymorphism has exhibited a noticeable association with weight, body length and chest circumference gains, in addition to the average daily gain in goats (Latifah et al., 2018). Likewise, several reports have also indicated a remarkable role of *MC4R* gene in live weight, backfat thickness, carcass traits and marbling score in several breeds of cattle (Lee et al., 2013; Liu et al., 2010; Seong et al., 2012). Additionally, genotyping studies of the *MC4R* gene have revealed a significant association of this gene with the milk yield and fat percentage in buffalos (Deng et al., 2016). Moreover, the *MC4R* gene polymorphism has been associated with backfat thickness in sheep (Zuo et al., 2014). The combined effect of multiple mutation sites within *MC4R* gene has also been included as a valuable factor for assessing its effect on economic traits (El‐Sabrout & Aggag, 2018). However, to accommodate such haplotyping studies, it is necessary to assess multiple genetic loci simultaneously (Lu et al., 2003).

Taking into consideration the above‐stated data, the combining of the possible interaction between *MC4R* polymorphism in Awassi and Arabi breed with live body weight and hormonal assays may have some potential in the detection of the possible correlation between both patterns.

Awassi, followed by Arabi, is the most predominant one in the middle and southern portions of Iraq (Al‐Shuhaib et al., 2019). Though Awassi sheep have well‐known living adaptation capacity, it has recently reported that Arabi sheep showed a higher genetic capability than Awassi sheep to cope up with harsh circumstances (Al‐Thuwaini et al., 2020). Furthermore, it has recently been stated that genetic diversity is considerably correlated with the productive performance in both breeds (Aljubouri & Al‐Shuhaib, 2020). Taking these genetic differences altogether, it is crucial to discriminate between both breeds to broaden our knowledge in critical production traits. Several biological functions have been associated with such adaptation conditions in sheep, such as live body weight and hormonal secretion (Marai et al., 2007). Thus, it is consequential to measure these values in both breeds taking advantage of the highly dynamic strategy sheep use to enhance their survival chances in harsh conditions (Niyas et al., 2015). Thus, it would be expected that Awassi and Arabi breeds could also exhibit different genetic variations in the *MC4R* gene in such a way it could affect functions associated with production traits. Therefore, the current study aims to describe the possible association between the *MC4R* gene polymorphism and the physiological differences between Awassi and Arabi breeds. Thus, single nucleotide polymorphisms (SNPs) of *MC4R* gene were identified, and association with live body weight was investigated to acquire possible molecular markers related to production traits for marker‐assisted selection. According to our knowledge, no report has screened the association of the coding sequences of the *MC4R* gene with live body weight and hormonal assays in sheep. Therefore, this research is the first one to describe such association in two breeds of sheep.

## 2 MATERIALS AND METHODS

### 2.1 Sheep population and ethical approval process

The study was conducted according to the international recommendations for the care and use of animals (Federation of Animal Science Societies, ), and the animal experimentations were approved by the Al‐Qasim Green University (Approval No. 12.10.18). A total of 150 sheep (*Ovis aries*) were selected randomly from three stations for raising sheep (Babylon, Karbala and Kufa provinces, Iraq) from January 2018 to August 2018. The three stations were approximately situated at a longitude of 32.6027°N, the latitude of 44.0197°E and 32 m a.s.l. Expert veterinarians confirmed the included sheep to be clinically healthy. Two types of breeds, including Awassi (*n* = 75, 22 rams and 53 ewes) and Arabi (*n* = 75, 15 rams and 60 ewes), were included in this study. Both included breeds were aged between 2.5 and 3 years. All included ewes were in non‐pregnant and non‐lactation status, and the parity of ewes was one to two parities. The animals of both breeds had different genetic and geographical backgrounds (Al‐Thuwaini et al., 2020). Awassi breed prevails in the Middle Euphrates regions with summer temperatures of <50°C, while Arabi breed prevails in Southern portions of Iraq with summer temperatures exceeding 50°C. Both breeds are fat‐tailed, carpet‐wool producers, with some potential to produce milk. However, both breeds had slightly different head appearance and body characteristics (Alkass & Juma, 2005). Animals were fed ad libitum on seasonal grass during summer, while in winter, animals were kept indoors and fed with a concentrated mixture consisting of barley grain (59%), bran (40%) and salt (1%). The live body weight of the sheep was recorded in the morning before the animals were grazing using a suspended spring balance, while blood tests were assessed. The descriptive statistics of estimated traits are shown in Table 1.

**TABLE 1:** Descriptive statistics of estimated traits used in the association analysis

Breed	Phenotypic traits	*N*	Mean	*SD*	Minimum	Maximum
A) Awassi						
	Live body weight (Kg)	75	41.909	4.113	25.0	52.0
	Estradiol (pg/ml)	75	28.980	9.272	12.040	67.148
	Testosterone (ng/ml)	75	0.928	0.035	0.107	4.666
B) Arabi						
	Live body weight (Kg)	75	37.650	2.225	25.0	48.0
	Estradiol (pg/ml)	75	25.915	10.174	10.630	51.869
	Testosterone (ng/ml)	75	0.711	0.079	0.147	4.185

Abbreviation: *N*, number of observation; *SD*, standard deviation.

### 2.2 Hormonal assay

Within 20 min of blood collection from the jugular vein, plasma was separated from peripheral blood by centrifugation at 3,500 g at 4°C for 15 min. Then, the plasma from each sample was collected and stored at –20°C. To assess the possible association between *MC4R* gene and sex hormones in both investigated breeds, testosterone and estradiol were measured using enzyme‐linked immunosorbent assay (ELISA) kit based on the sandwich principle (cat. no. E0013Sh for testosterone and cat. no. E0047 Sh for estradiol, Bioassay Technology Laboratory Co.). The absorbance of hormones was measured at 450 nm using a microplate reader (ELx808 Ultraplate Reader, BioTek Instruments, Inc.). Concentrations were presented as pg/ml and ng/ml for estradiol and testosterone, respectively. A standard curve was generated, and samples were interloped according to the manufacturer's instructions (Bioassay Technology Laboratory Co.).

### 2.3 Genomic DNA extraction, primer design and PCR

**FIGURE 1:**
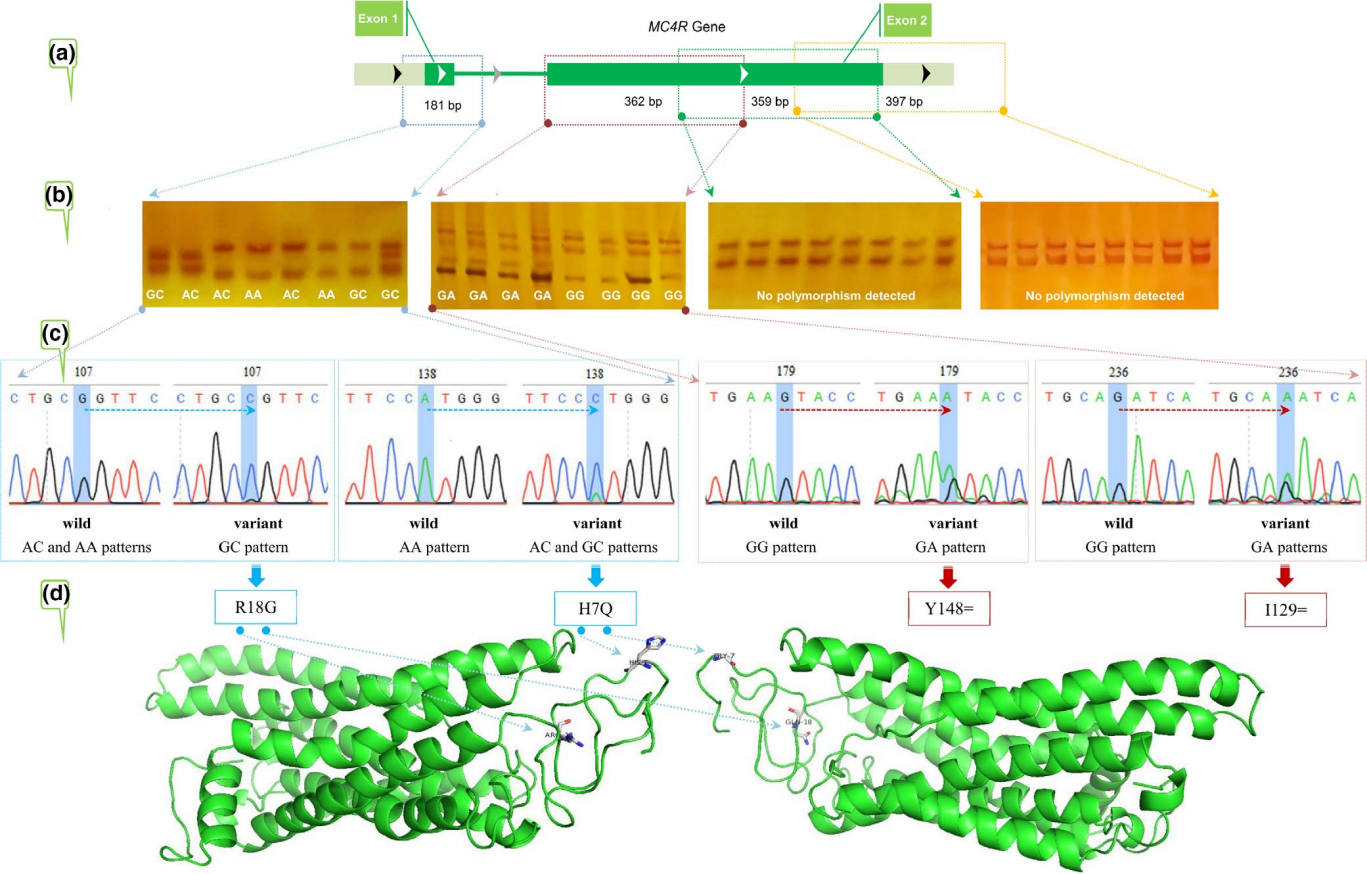
A schematic diagram of the present study to assess the *MC4R* gene polymorphism in the Awassi and Arabi sheep. (a) The exact genomic positions of the amplify a portion of the ovine *MC4R* gene were described according to GenBank acc. no. NC_019480.2. (b) PCR‐single‐strand conformation polymorphism (PCR‐SSCP) genotyping of the amplified loci. (c) DNA sequencing chromatogram of the polymorphic fragment. (d) The *in silico* prediction of *MC4R* SNPs

Genomic DNA was isolated from the whole blood using a rapid and efficient salting‐out method (Al‐Shuhaib, 2017). Four pairs of specific polymerase chain reaction (PCR) oligonucleotides were designed to cover all the coding sequences of the ovine *MC4R* gene using NCBI Primer Blast online server (Ye et al., 2012). For exon 1, only one PCR primer pair was designed, while three pairs of primers were designed to adequately cover the coding sequences in the exon 2 (Figure 1a). The lyophilized oligonucleotides were purchased from Bioneer Company (Bioneer, Korea). The PCR primers were designed based on GenBank accession no. NC_019480.2 and the details of the primer used in this study are shown in Table 2. PCR experiments were conducted using *AccuPower^®^* PCR PreMix (Bioneer), and initiated by denaturation for 5 min, followed by 30 cycles (annealing at 57.8–59.1°C for 30 s each), and finalized with polymerase extension (72°C) for 5 min. The target amplified PCR products were confirmed by 1.5% agarose gel electrophoresis and then submitted to single‐strand conformation polymorphism (SSCP) protocols (Hashim & Al‐Shuhaib, 2019).

**TABLE 2:** Oligonucleotide primer sets designed for the amplification of the *MC4R* in Awassi and Arabi sheep population. The present annotations of this study variants were based on GenBank accession number NC_019480.2

Set	Primer code	Primer sequence (5′→3′)	Length	Annealing temp.
1	MC4R,exo1‐F	GTCACAAACACCTCGGGAGA	181 bp	57.8°C
	MC4R,exo1‐R	TCCAGAGGGGACCTGAATCC		
2	MC4R,exo2,1‐F	TGGGGGCAGGAGATGTAGAA	359 bp	57.8°C
	MC4R,exo2,1‐R	GCGCTCCAGTACCATAGCAT		
3	MC4R,exo2,2‐F	TGAGAGCCAGCATGGTGAAG	362 bp	60.4°C
	MC4R,exo2,2‐R	TGTGGCTGACATGTTGGTGA		
4	MC4R,exo2,3‐F	ACCGCAGTTTGTCCCCATTT	397 bp	59.1°C
	MC4R,exo2,3‐R	CATGGCGTCTCTCTACGTCC		

### 2.4 Single‐strand conformation polymorphism (SSCP) and sequencing analysis

The initial denaturation of the PCR products, as well as SSCP protocol, was performed according to Al‐Shuhaib et al., (2018) with some optimization suitable for the designed amplicons. Briefly, SSCP analyses were conducted in polyacrylamide gels (37.5:1) with TBE (0.5X) buffer at a constant temperature of 10°C using 216 × 110 mm mini‐wide gels with 1 mm gel thickness (JY‐CZ‐B, Junyi‐Dongfang Electrophoresis Equipment). Gel concentrations were adjusted to 10% and 8% for exon 1 and exon 2 amplicons, respectively. Electrophoresis conditions applied were 210 V/105 mA/5 hr and 200 V/100 mA/4 hr for exon 1 and exon 2 amplicons, respectively. Bands were fixed and stained according to the protocol described by Byun et al., (2009). Each detected SSCP‐banding pattern of all investigated samples was sent for sequencing from both termini according to the instruction manual described by Macrogen laboratories (Geumcheon). The electropherograms were edited and aligned using EditSeq software, ver. 7.1.0 (DNASTAR, Lasergene). The observed mutations were visualized and annotated using SnapGene Viewer, ver. 4.0.4. (GSL. Biotech. LLC). The novelty of the observed variants was checked by exploring variants of the ovine *MC4R* gene database in the Ensembl genome browser 96 (https://asia.ensembl.org/index.html).

### 2.5 *In silico* prediction

Several computational tools were utilized to assess the consequences of the observed missense variants on the resulting mutant protein structures and functions, namely SIFT (Ng & Henikoff, 2003), PolyPhen‐2 (Adzhubei et al., 2010), PROVEAN (Choi et al., 2012), Panther (Tang & Thomas, 2016) and PhD SNP (Capriotti et al., 2006). The 3D structure of *MC4R* was generated by RaptorX server before and after mutation (Källberg et al., 2012), and validated in verify3D and PROCHECK servers (http://servicesn.mbi.ucla.edu/Verify3D/).

### 2.6 Genetic polymorphism and statistical analyses

The allele and genotype frequencies, observed heterozygosity (*Ho*), expected heterozygosity (*He*) and an effective number of alleles were analysed using PopGen32 software, ver. 1.31 (Yeh & Yang, 1999). The polymorphism information content (*PIC*) was calculated by utilizing the HET software ver. 1.8 (Ott, 2001). Pairwise linkage disequilibrium (LD) between SNPs was calculated by *r* ^2^ and *D*' values using SHEsis software (She & He, 2006).

The significant effects of breed, sex and SNP genotype on the various studied parameters were analysed by SPSS software ver. 23.0 (SPSS Inc), using the general linear model:

$$y_{\mathrm{ijkl}}=\mu +B_{i}+S_{j}+G_{k}+e_{\mathrm{ijkl}},$$

where *y_ijkl_* is phenotypic traits, *μ* is the overall mean, *B_i_* is the fixed effect of ith breed (*i* = Awassi, Arabi), *S_j_* is the fixed effect of jth sex (*j* = ram, ewe), *i_k_* is the fixed effect of *k*th SNP genotype or combined genotype and *e_ijkl_* is the random error associated with *y_ijkl_* observation and assumed to be NID (0, σ2e). Means were compared using the Tukey‐Kramer test with a significance level of *p* < .05. Preliminary statistical analysis indicated that the effects of age, parity, season, station and the interaction between these effects were not included in the final model because they did not have a significant effect on phenotypic traits.

### 2.7 SNP genotype effects estimation

For the SNP that showed significant association with the phenotypic traits, differences between the means of each genotype and allelic frequencies were used to estimate additive effects (Hill & Mackay, 2004). The following formula was utilized to find additive genetic variance (Var*_A_*) imputed to a SNP:

$${\text{Var}}_{A}=2p_{i}q_{i}\alpha_{i}^{2},$$

where *q* and *p* were the allelic frequencies for the *j*th SNP predicted across the entire population, *α_i_* – SNP allele substitution effect obtained from a linear regression model in a statistical program, in which the genotypes recorded as a variate of 0, 1 and 2 copies of a particular allele.

The proportion of the phenotypic variance explained by SNP(s) was calculated by (%) var*P* = 100 × 2*p_i_q_i_* $\alpha_{i}^{2}$/*V_P_*, where *p* and *q* denote the SNP allele frequency; $\alpha_{i}^{2}$ is the SNP allele substitution effect, 2*p_i_q_i_* $\alpha_{i}^{2}$ is the additive genetic variance and *V_P_* is the phenotypic variance.

## 3 RESULTS

### 3.1 The genetic polymorphism and *in silico* analysis

Four amplicons were used to scan all the coding regions of the *MC4R* gene, including only one amplicon (181 bp) for exon 1, and three amplicons (362 bp, 359 bp and 397 bp) for the exon 2. Only exon 1 amplicons showed heterogeneity with a missense effect, while the exon 2 (362 bp) amplicons showed heterogeneity with a silent mutation. However, only monomorphous SSCP‐banding patterns were revealed in both 359 and 397 bp amplicons. Thus, the genotyping by the SSCP method was utilized in exon 1 to assess the pattern of genetic variation in both breeds. Three SSCP‐banding patterns were detected in exon 1 in the same SSCP gel conditions (Figure 1b). Sequencing reactions confirmed these patterns and allowed the identification of 10 SNPs in exon 1 with missense and silent effects. Eight of these SNPs, 102G > C (S19R), 113A > C (W16G), 117G > A (H14=), 122G > A (L13F), 124G > A (S12F), 126G > A (T11=), 153G > A (N2=) and 168A > C (N2=), were detected in all analysed samples, while two SNPs, 138A > C (H7Q) and 107G > C (R18G), exhibited variable distributions in the exon 1. Both 107G > C and 138A > C SNPs were found to be responsible for the observed heterogeneity of samples having GC and AC genotypes, respectively. The other samples with a homologous SSCP‐banding pattern (AA genotype) did not exhibit any detectable unique variation. The 107G > C SNP was found to cause the amino acid R18G (arginine to glycine) substitution. Whereas the (H7Q) histidine to glutamine amino acid change was caused by the 138A > C SNP (Figure 1c). Concerning exon 2, both PCR‐SSCP method as well as sequencing reactions revealed a remarkably less polymorphic status than the exon 1. No polymorphism was detected in both 359 bp and 397 bp amplicons, while two different SSCP‐banding patterns were identified in the 362 bp amplicons. Sequencing reactions confirmed the presence of two SSCP patterns in the 362 bp amplicons with the detection of two SNPs in analysed samples with GA genotype (179G > A and 236G > A), while no polymorphism was observed in samples with GG genotype.

A series of computational tools were utilized to assess the final consequences of this missense variant on the altered MC4R protein in structure and function, such as SIFT, PolyPhen‐2, PROVEAN, Panther and PhD SNP (Table 3). All of these tools lead to neutral/non‐deleterious signals for both assessed R18G and H7Q. This non‐deleterious effect may be due to their non‐critical positions in the generated 3D structure of *MC4R* protein (Figure 1d).

**TABLE 3:** *In silico* prediction of H7Q and R18G on ovine MC4R protein, in terms of structure and function

Tool	H7Q		R18G	
	Score	Prediction	Score	Prediction
SIFT	0.44	Tolerated	0.24	Tolerated
PolyPhen‐2	0.148	Benign	0.132	Benign
PROVEAN	−0.042	Neutral	0.24	Neutral
PANTHER	Unknown preservation time	Invalid substitution	30 (preservation time)	Probably benign
PhD SNP	89% confidence	Neutral	78% confidence	Neutral

### 3.2 Assessment of *MC4R* polymorphism and association analysis

**FIGURE 2:**
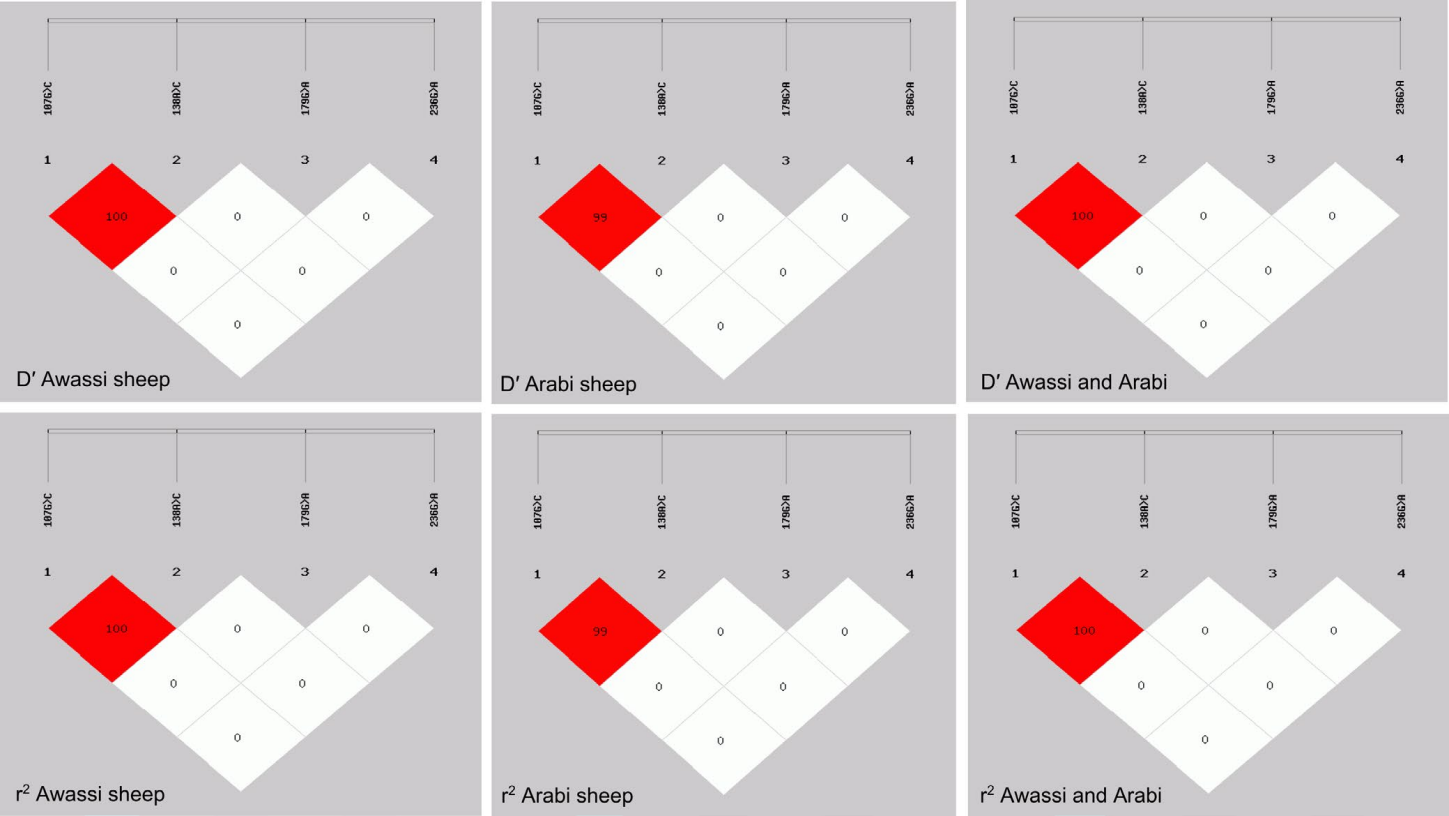
Linkage disequilibrium plot of the observed four SNPs within the exon 1 and exon 2 amplicons of the ovine *MC4R* gene in Awassi, Arabi and two breeds. (a) *D'* value, (b) *r* ^2^ value. The red diamonds indicate strong LD between pairs of SNPs

Genotype, allele frequencies and genetic diversity parameters for exon 1 amplicon in the analysed populations of Awassi and Arabi sheep are presented in Table 4, A and B. According to the value of Chi‐square, the population under study was not in Hardy–Weinberg equilibrium (HWE), which was statistically significant at *p* ˂ .05. According to the classification of *PIC* (low polymorphism, if *PIC* value < 0.25; median polymorphism if 0.25 < *PIC* value < 0.5 and high polymorphism if *PIC* value > 0.5; Trakovická et al., 2013), analyses showed that four SNPs (107G > C, 138A > C, 179G > A and 236G > A) were considered as moderate *PIC*. Pairwise LD between SNPs was calculated and indicated that the D' and r^2^ values were between 0.99 and 1.00, signifying a close connection and strong co‐inheritance for both 107 G > C and 138 A > C in both breeds (Figure 2). The haplotypes of the two SNPs were constructed for each breed and in all analysed populations. In relative terms, there was no LD between the two SNPs (179G > A and 236G > A) in exon 2 also with SNP pairs in exon 1.

**TABLE 4:** Genotype, allele frequencies and genetic diversity parameters in the *MC4R* gene for both Awassi and Arabi breeds

	SNP	Genotypes (*N*)	Genotype frequencies	Allele	Allele frequencies	*χ* ^2^	*Ho*	*He*	*Ne*	*PIC*
A) Arabi	179G > A	GG(37) GA(38)	0.49 0.51	G A	0.75 0.25	8.362*	0.506	0.380	1.608	0.304
	236G > A	GG(41) GA(34)	0.55 0.45	G A	0.77 0.23	6.223*	0.453	0.352	1.539	0.291
	107G > C 138A > C	GG/AA(7) GC/AC(42) CC/CC(26)	0.09 0.56 0.35	GA —	0.37 —	55. 733*	0.906	0.629	2.667	0.357
				CC	0.63					
B) Awassi	179G > A	GG(42) GA(33)	0.56 0.44	G A	0.78 0.22	5.757*	0.440	0.345	1.522	0.284
	236G > A	GG(39) GA(36)	0.52 0.48	G A	0.76 0.24	7.238*	0.480	0.367	1.574	0.298
	107G > C 138A > C	GG/AA(12) GC/AC(45) CC/CC(18)	0.16 0.60 0.24	GA —	0.46 —	46.127*	0.840	0.601	2.485	0.373
				CC	0.54					

Abbreviations: He, Expected heterozygosity;Ho, observed heterozygosity; *Ne*, effective allele number; PIC, polymorphism information content; χ^2^, Chi‐square.

*All Chi‐square tests have two degrees of freedom and within the significance level *p* < .05.

Awassi breed showed higher live body weight (44.855 ± 0.683) in comparison to Arabi breed (38.333 ± 0.790; *p* < .05). Meanwhile, the conducted sex hormone assays showed no significant (*p* > .05) differences (0.618 ± 0.005 ng/ml and 0.987 ± 0.003 ng/ml for testosterone, and 26.826 ± 1.966 pg/ml and 29.800 ± 3.296 ng/ml for estradiol) between Arabi and Awassi breeds, respectively. Association analyses for live body weight and sex hormone assays according to the breed and sex effect are presented in Table 5. These results indicated that Awassi breed had more body weight gain than Arabi breed. In regards to sex effect, significant differences (*p* < .05) between rams and ewes were identified, being heavier weight in the ewes (39.833 ± 1.328) in comparison to rams (34.167 ± 0.511). Based on the haplotype analysis, a combined analysis of genotypes revealed that the haplotype block was significantly associated with phenotypic traits (*p* < .05). The sheep with CC/CC haplotype showed higher live body weight (48.567 ± 2.956; 45.855 ± 3.626; 47.231 ± 1.230) and lower estradiol level (16.745 ± 1.460; 14.410 ± 1.883; 15.577 ± 1.266) in Awassi, Arabi and both breeds, respectively, than the other combined genotypes. Whereas the testosterone level showed no significant differences among the observed haplotypes (*p* > .05). Individuals with GC/AC haplotype showed higher live body weight (41.324 ± 3.961; 33.176 ± 2.581; 37.250 ± 0.790) than those with GG/AA haplotype (30.800 ± 2.347; 29.696 ± 2.899; 30.244 ± 0.968; *p* < .05) in Awassi, Arabi and both breeds, respectively. Whereas no significant differences (*p* > .05) were observed in the concentrations of estradiol and testosterone between GG/AA and GC/AC haplotypes. These results indicated that the individuals with GC/AC haplotype had a higher body weight and could be used in the improvement of growth traits in Awassi and Arabi breeds, respectively. Furthermore, the genotype effect prediction confirmed that the GC/AC haplotype was associated with higher body weight (*p* < .05). Greater genetic variance percentage with the phenotypic traits (>1%) is detailed in Table 6A and B. Meanwhile, the individuals with CC/CC haplotype had higher live body weight with a lower level of estradiol indicating that this haplotype should not be extensively selected in sheep breeding for litter size in Awassi breed. However, the association analysis in exon 2 showed no significant difference (*p* > .05) between 179G > A and 236G > A and phenotypic traits.

**TABLE 5:** Least square means ± *SE* for live body weight and sex hormone assay in association with the *MC4R* polymorphism in Awassi (A), Arabi (B) and both breeds (C)

Breed	Locus	Genotype	Live body weight (Kg)	Testosterone (ng/ml)	Estradiol (pg/ml)
A) Awassi	Exon 1;107G > C	GG/AA	30.800 ± 2.347^c^	1.306 ± 0.036^a^	25.109 ± 3.972^a^
	138A > C	GC/AC	41.324 ± 3.961^a^	0.869 ± 0.029^a^	22.101 ± 2.725^a^
		CC/CC	48.567 ± 2.956^b^	0.613 ± 0.057^a^	16.745 ± 1.460^b^
	Exon 2;179G > A	GG	29.191 ± 2.725^c^	0.920 ± 0.036^a^	22.203 ± 2.809^a^
	236G > A	GA	31.400 ± 3.460^c^	1.120 ± 0.029^a^	22.635 ± 3.388^a^
B) Arabi	Exon 1;107G > C	GG/AA	29.696 ± 2.899^c^	1.106 ± 0.027^a^	24.149 ± 2.010^a^
	138A > C	GC/AC	33.176 ± 2.581^a^	0.860 ± 0.010^a^	20.098 ± 3.250^a^
		CC/CC	45.855 ± 3.626^b^	0.534 ± 0.024^a^	14.410 ± 1.883^b^
	Exon 2;179G > A	GG	31.062 ± 2.167^c^	0.923 ± 0.057^a^	22.218 ± 2.972^a^
	236G > A	GA	31.040 ± 2.081^c^	0.922 ± 0.035^a^	20.408 ± 2.756^a^
C) Awassi and Arabi	Exon 1;107G > C	GG/AA	30.244 ± 0.968^c^	1.204 ± 0.004^a^	24.627 ± 2.870^a^
	138A > C	GC/AC	37.250 ± 0.790^a^	0.865 ± 0.014^a^	21.098 ± 2.870^a^
		CC/CC	47.231 ± 1.230^b^	0.574 ± 0.003^a^	15.577 ± 1.266^b^
	Exon 2;179G > A	GG	30.123 ± 0.526^c^	0.921 ± 0.001^a^	22.210 ± 1.320^a^
	236G > A	GA	31.220 ± 0.466^c^	1.021 ± 0.002^a^	21.521 ± 4.421^a^

Note

Different superscript in the same column within each classification indicates significant differences (*p* < .05).

Different superscript a, b, c indicates the significant difference.

**TABLE 6:** Effect of substitution in 107G > C and 138A > C polymorphism of *MC4R* on the live body weight and estradiol hormone of Awassi and Arabi breed. (A) SNP allele substitution effect (α) is shown. (B) Haplotype (h) substitution effect is shown

A)	Phenotypic traits	SNPs	Substitution allele	α Allele substitution effect	var*P* (%)
Awassi	Live body weight (Kg)	107G > C	G	1.821	3.315
	Estradiol (pg/ml)	107G > C	G	1.742	1.741
	Live body weight (Kg)	138A > C	A	1.952	3.808
	Estradiol (pg/ml)	138A > C	A	1.724	2.971
Arabi	Live body weight (Kg)	107G > C	G	1.573	2.473
	Estradiol (pg/ml)	107G > C	G	1.421	2.018
	Live body weight (Kg)	138A > C	A	1.601	2.561
	Estradiol (pg/ml)	138A > C	A	1.324	1.752

B)	Phenotypic traits	SNPs	Haplotype	*h* substitution effect	var*P* (%)
Awassi	Live body weight (Kg)	107G > C + 138A > C	GG/AA	1.930	3.723
	Estradiol (pg/ml)	107G > C + 138A > C	GG/AA	1.724	2.971
	Live body weight (Kg)	107G > C + 138A > C	GC/AC	1.952	3.808
	Estradiol (pg/ml)	107G > C + 138A > C	GC/AC	1.742	3.033
	Live body weight (Kg)	107G > C + 138A > C	CC/CC	1.992	3.967
	Estradiol (pg/ml)	107G > C + 138A > C	CC/CC	1.821	3.315
Arabi	Live body weight (Kg)	107G > C + 138A > C	GG/AA	1.853	3.432
	Estradiol (pg/ml)	107G > C + 138A > C	GG/AA	1.830	3.348
	Live body weight (Kg)	107G > C + 138A > C	GC/AC	1.873	3.507
	Estradiol (pg/ml)	107G > C + 138A > C	GC/AC	1.850	3.421
	Live body weight (Kg)	107G > C + 138A > C	CC/CC	1.923	3.695
	Estradiol (pg/ml)	107G > C + 138A > C	CC/CC	1.911	3.650

Note

var*P* proportion of phenotypic variance explained. The percentage of variance was calculated only for the SNPs that showed a significant association with the traits.

## 4 DISCUSSION

Association analyses for live body weight revealed that the Awassi breed showed higher live body weight in comparison to Arabi breed, indicating that Awassi breed had more body weight gain than Arabi breed. The breed of sheep was known to have an important impact on body weight (Aktaş et al., 2015). Awassi sheep were shown to respond favourably to body weight than other breeds (Galal et al., 2008). In regards to sex effect, this study found that ewes showed heavier weight compared to rams in both breeds. These differences might be attributed to the physiological and sexual hormones of rams and ewes (Kratochvílová et al., 2002). Indeed, oestrogen hormone is stimulating specific receptors in the pro‐opiomelanocortin (POMC) neurons in the hypothalamus by the melanocortin system (Clegg et al., 2006). It has been reported that increased POMC levels result in more binding of α‐MSH to *MC4R*, promoting increased food intake and reduced energy expenditure in ewes (El‐Sabrout & Soliman, 2018; Hewagalamulage et al., 2015). Furthermore, females are more severely affected than males in weight increase (Vaisse et al., 2000).

The *MC4R* gene/protein variation is a key component in the *MC4R* pathway, which regulates phenotypic traits. Sequencing analyses of the current study confirmed the identification of two missense SNPs, 107G > C and 138A > C, in exon 1 of the *MC4R* gene. Both identified SNPs were associated with live body weight and sex hormone levels. El‐Sabrout (2017) found that the SNPs of the *MC4R* gene were associated with body weight and sexual desire behaviour in rabbits. Our results are consistent with numerous studies conducted in livestock species that reported the association of the *MC4R* genetic polymorphism with phenotypic traits. Individuals with a synonymous 93G > A mutation in the *MC4R* gene have significantly associated with a backfat thickness in sheep (Zuo et al., 2014). The g.998A/G SNP was significantly associated with weight gain, body length and chest circumference gain in Bligon goats (Latifah et al., 2018). Add to that, individuals with the AG and GG genotypes (−129A > G) in the *MC4R* gene had higher live body weight than individuals with AA genotype in cattle (Liu et al., 2010). In another study, individuals with heterozygous genotype at g.1104C > T of the *MC4R* gene showed better performance than individuals with homozygous genotype for milk yield in Chinese buffaloes (Deng et al., 2016). Although the association study between single SNP with phenotypic traits is likely to be easier and more efficient in breeding programs, the haplotype analysis is valuable for assessing the effect of genes on phenotypic traits. In this study, statistical analysis showed that individuals with GC/AC haplotype were more associated with live body weight gain than those with GG/AA and CC/CC haplotypes, respectively. These results confirmed that the priority of genomic and inbreed selection should be held on heterozygous states compared to homozygous ones. The study of Cai et al., (2015) showed that individuals with CGACG and CTCCC haplotypes of the *MC4R* gene were associated with an increased body weight of animals aged 18 months in yaks. Based on previous information, Awassi sheep is a low prolific breed (Abdullah et al., 2002), and very low incidence of twinning (Al‐Sa'aidi et al., 2018). The present study demonstrated that individuals with CC/CC haplotype had higher live body weight and lower levels of estradiol, which indicates that this haplotype has not been extensively selected in sheep breeding to enhance litter size and twinning ratio in Awassi breed. This fact was confirmed by Chen et al., (2017) who reported that the variation of *MC4R* gene implies a reduction of GnRH–LH secretion and dysfunction of the ovary in mice, resulting in a reduction in the number of developed corpora lutea and decreased litter sizes.

## 5 CONCLUSION

This study identified two co‐inherited novel SNPs (107G/C and 138 A/C) of *MC4R* gene in two breeds of sheep. Both of the 107G/C and 138A/C SNPs were found to be highly associated with live body weight and hormonal (testosterone and estradiol) assays. We demonstrated that the GC/AC haplotype affects the sheep live body weight and hormonal assays and it is therefore highly recommended to be selected and fixed for sheep production.

## CONFLICT OF INTEREST

The authors declare that they have no conflict of interest.

## AUTHOR CONTRIBUTIONS

**Tahreer M. Al‐Thuwaini**: Conceptualization; supervision; Methodology (lead). **Mohammed Baqur S. Al‐Shuhaib**: Writing‐original draft, editing, Data curation and investigation (lead). **Frederic Lepretre**: Data curation and investigation (equal). **Halla Hassan Dawud**: Methodology (equal).

## Funding information

The authors have not declared a specific grant for this research from any funding agency in the public, commercial or not‐for‐profit sectors.

## ETHICAL APPROVAL

The study was conducted according to international recommendations for the care and use of animals (Federation of Animal Science Societies, 2010) and the animal experimentations were approved by Al‐Qasim Green University (Approval No. 12.10.18).

### Peer Review

The peer review history for this article is available at https://publons.com/publon/10.1002/vms3.421.
